# (*E*,*E*)-3-Methyl-2,5-bis­(4-methyl­benzyl­idene)cyclo­penta­none: synthesis, characterization, Hirshfeld surface analysis and anti­bacterial activity

**DOI:** 10.1107/S2056989019003827

**Published:** 2019-03-26

**Authors:** Fatiha Mahdi, Assia Sid, Rafika Bouchene, Paul Mosset, Thierry Roisnel

**Affiliations:** a Laboratoire des Sciences Analytiques, Matériaux et Environnement (LSAME), Université Larbi Ben M’hidi, Oum El Bouaghi, 04000, Algeria; b Département Sciences de la Matière, Faculté des Sciences Exactes et Sciences de la Nature et de la Vie, Université Larbi Ben M’hidi, Oum El Bouaghi, 04000, Algeria; c Université de Rennes 1, Institut des Sciences Chimiques de Rennes, CNRS UMR, 6226, Avenue du Général Leclerc, 35042 Rennes Cedex, France

**Keywords:** Claisen–Schmidt reaction, crossed-aldol condensation, crystal structure, spectroscopic studies

## Abstract

The title compound, (*E*,*E*)-3-methyl-2,5-bis­(4-methyl­benzyl­idene)cyclo­penta­none (MBMCP), C_22_H_22_O, was obtained by Claisen–Schmidt condensation of 4-methyl­benzaldehyde with 3-methyl­cyclo­penta­none in good yield. The structure of MBMCP was studied using UV, FT–IR and Raman spectroscopy, single-crystal X-ray diffraction (XRD) measurements, and ^1^H and ^13^C nuclear magnetic resonance (NMR) spectroscopy. The mol­ecular structure of MBMCP is fully extended in the *E*,*E* configuration. C—H⋯π stacking inter­actions play a significant role in the stabilization of the mol­ecular packing. Hirshfeld surface analysis was used to qu­antify the non-covalent inter­actions in the crystal lattice. Microbiological studies were performed to investigate the anti­microbial activity of this new product.

## Abstract 2

The title compound has been synthesized by a Claisen–Schmidt reaction. The mol­ecular structure is fully extended in the *E*,*E* configuration. C—H⋯π inter­actions have a dominant role among the inter­molecular inter­actions.

## Chemical context   

The Claisen–Schmidt reaction has great importance in the synthesis of organic compounds (Rajput & Kaur, 2012 ▸), in particular in the synthesis of bis­(substituted-benzyl­idene)cyclo­alkanones. This reaction is catalysed using strong acids (Dhar & Barton, 1981 ▸; Gall *et al.*, 1999 ▸) and base with or without solvents (Geissman & Clinton, 1946 ▸; Shan *et al.*, 2010 ▸). Recently, animal bone meal was used as a catalyst for crossed-aldol condensation (Riadi *et al.*, 2010 ▸). The preparation of β-chloro, β-bromo and α,β-unsaturated ketones from β-di­ketones has been carried out using Vilsmeier reagents (Mewshaw *et al.*, 1989 ▸). Numerous *α*,*α*′-bis­(substituted-benzyl­idene)cyclo­alkanones exhibit biological activities (Robinson *et al.*, 2005 ▸; Piantadosi *et al.*, 1973 ▸). Furthermore, they are used as precursors for the preparation of biologically active heterocyclic compounds, such as pyrimidines (Deli *et al.*, 1984 ▸; Guilford *et al.*, 1999 ▸) and pyrazolines (Ziani *et al.*, 2013 ▸). These compounds have received a lot of attention because of their uses as perfume inter­mediates, pharmaceutical, agrochemical and liquid-crystal polymer units (Artico *et al.*, 1998 ▸; Amoozadeh *et al.*, 2010 ▸). *α*,*α*′-Bis(substituted-benz­yl­idene)cyclo­alkanones are also essential pharmacophores of various natural products (Shetty *et al.*, 2015 ▸). An example of permitted therapeutic agents including this mol­ecular framework is coumarin-chalcone (anti­cancer agents).

In the present work, we have synthesized, in one-step, (*E*,*E*)-3-methyl-2,5-bis­(4-methyl­benzyl­idene)cyclo­penta­none (MBMCP) by NaOH-catalysed Claisen–Schmidt condensation of 4-methyl benzaldehyde with 3-methyl cyclo­penta­none (see scheme). The structure of MBMCP was investigated by UV, FT–IR and Raman spectroscopy, single crystal X-ray diffraction (XRD) measurements and ^1^H and ^13^C nuclear magnetic resonance (NMR) spectroscopy.
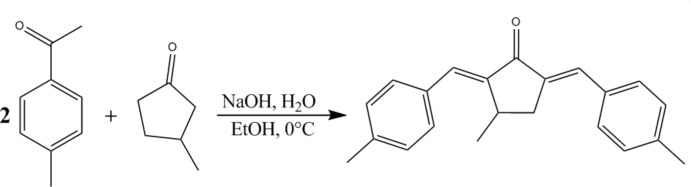


Several studies on the biological activity of unsaturated carbonyl compounds have been carried out. As an example, a series of chalcone derivatives that mimic the essential properties of cationic anti­microbial peptides were designed and synthesized by Chu *et al.* (2018 ▸). The anti­bacterial activities of these chalcones against drug-sensitive bacteria, including *Staphylococcus aureus*, *Enterococcus faecalis*, *Escherichia coli* and *Salmonella enterica* indicate that these compounds have potential therapeutic effects against bacterial infections. The phenyl group and the fluoride atom in these compounds were found to play an important role in their anti­bacterial and hemolytic activities. The above findings prompted us to evaluate the anti­bacterial activity of MBMCP *in vitro* against four bacterial strains**.**

## Structural commentary   

**Figure 1:**
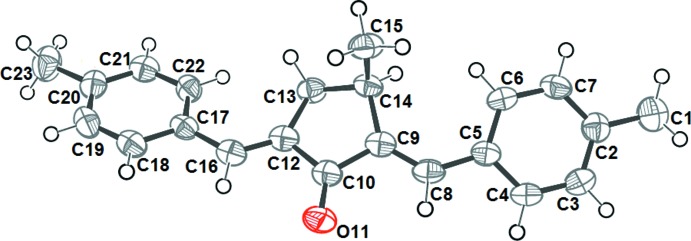
The asymmetric unit of MBMCP, showing the atom-numbering scheme. Displacement ellipsoids are drawn at the 50% probability level.

The structure of MBMCP was confirmed using single X-ray diffraction. The asymmetric unit comprised a single mol­ecule, illustrated in Fig. 1 ▸ with the atom-numbering scheme. The cyclo­penta­none ring adopts a half-chair form, with deviations of −0.146 (2) and 0.160 (2) Å from the mean plane of the ring for C13 and C14, respectively. The torsion angles within the five-membered ring are 4.7 (2) (C9—C10—C12—C13), −19.5 (2)° (C10—C12—C13—C14) and 26.3 (2)° (C12—C13—C14—C9), confirming the non-planarity of the central ring. The C15 methyl group in MBMCP lies practically perpendicular to the plane of its attached cyclo­penta­none ring, with torsion angle C12—C13—C14—C15 of −91.4 (2)°. The mean planes of the phenyl rings at either end of the mol­ecule are twisted with respect to one another by 41.3 (1)°.

The C8=C9 and C12=C16 bond lengths are 1.344 (3) and 1.335 (3) Å, respectively, and those for C8—C5 and C16—C17 are 1.464 (3) and 1.462 (3) Å, respectively. These values are in between the normal values for single and double bonds (1.54 and 1.33 Å, respectively), which is consistent with a delocal­ized π-bonding system.

The mol­ecular structure of MBMCP is fully extended in the *E*,*E* configuration stabilized by two short intra­molecular contacts, H8⋯O11 and H16⋯O11 (2.55 and 2.53 Å, respectively).

## Spectroscopic results   

The FT–IR spectrum of MBMCP shows the strong band of a conjugated carbonyl group at 1670 cm^−1^ and two bands at 1616 and at 1596 cm^−1^ for the two non-equivalent exo-cyclic C=C bonds. The Raman spectrum shows two characteristic bands in the 1720–1670 and 1620–1590 cm^−1^ regions, which indicate the presence of carbonyl groups conjugated with the double bonds.

The above result is also confirmed using the chemical shifts in the ^13^C NMR spectrum for the carbonyl groups (196.46 ppm) and of the C=C group at (139.7 and 139.8 ppm). The dienone of cyclic ketone derivatives occur in *E*,*E*, *Z*,*Z*, or *Z*,*E* configurations (Vatsadze *et al.*, 2006 ▸) and we have obtained the *E,E* isomer. The ^1^H NMR spectrum shows the signals of CH protons at a greater field than 7.2 ppm (δ = 7.51–7.53 ppm), which is in agreement with the *E* isomers, whereas the signals for the *Z* isomers are identified using the chemical shifts at δ ∼6.8 ppm (George & Roth, 1971 ▸).

The UV spectrum of the designated compound in ethanol reveals four absorption bands at 208 nm (∊ = 3.215 L mol^−1^cm^−1^), 237 nm (∊ = 1.984 L mol^−1^cm^−1^), 364 nm (∊ = 3.215 L mol^−1^cm^−1^) and 376 nm (∊ = 2.436 L mol^−1^cm^−1^) assigned to *n*–π, π–π* transitions.

## Supra­molecular features   

Mol­ecules of MBMCP pack with no classical hydrogen bonds. However, C18—H18⋯O11(1 − *x*, −*y*,1 − *z*) and C23—H23*B*⋯O11(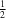 + *x*, 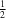 − *y*, 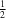 + *z*) short contacts occur, where the oxygen atom of the carbonyl group works as an acceptor with O11⋯H distances of 2.61 and 2.66 Å, respectively. These inter­actions are neglected as the H⋯O van der Waals distance is 2.60 Å and C—H⋯O contacts frequently have H⋯O separations shorter than 2.4 Å (Taylor & Kennard, 1982 ▸). On the other hand, even though the carbonyl group is a strong acceptor, the O atom acts as a multiple acceptor. This condition corresponds to an important argument for the structural importance of the C—H⋯O hydrogen bond (Steiner, 1996 ▸).

**Figure 2:**
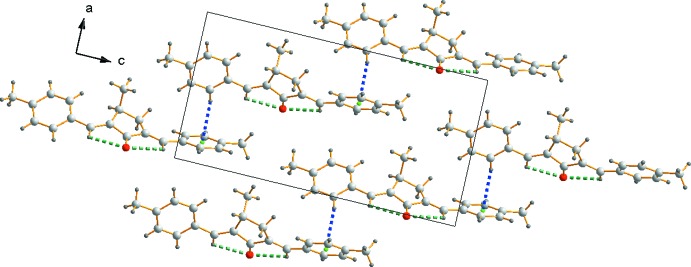
The one-dimensional chain structure of MBMCP formed *via* C—H⋯π inter­actions (blue dashed lines). The green spheres indicate the centroids of the phenyl ring.

Mol­ecular chains of MBMCP propagate along the [101] direction through a C—H⋯π inter­action (Table 1 ▸) involving the C4–H4 group of the C2–C7 phenyl ring pointing towards the π cloud on an adjacent C17–C22 ring, as shown in Fig. 2 ▸. The contact distances are consistent with those of the C—H⋯π edge-to-face inter­actions observed in the crystal structure of benzene (Bacon *et al.*, 1964 ▸).

**Figure 3:**
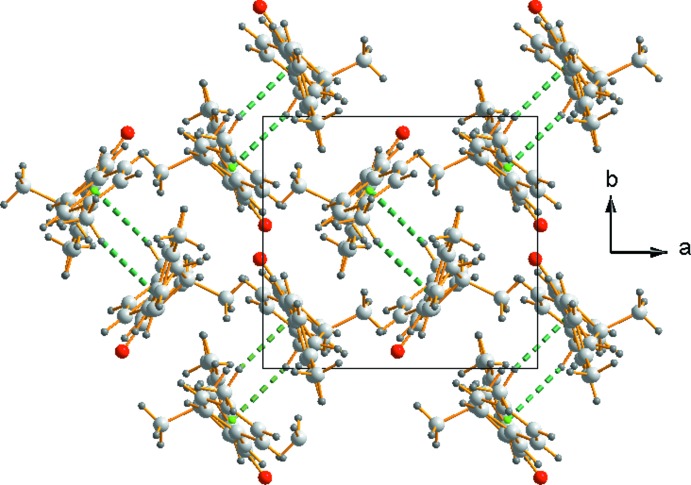
Crystal packing of MBMCP. The green spheres indicate the centroids of the phenyl ring.

The mol­ecules of MBMCP stack in waves along the *a-*axis direction, as shown in Fig. 3 ▸. The methyl carbon (C1) makes an important contribution to the stability of this stacking arrangement *via* the establishment of a C—H⋯π inter­action with the centroid of a neighboring aryl ring.

## Hirshfeld surface analysis   

**Figure 4:**
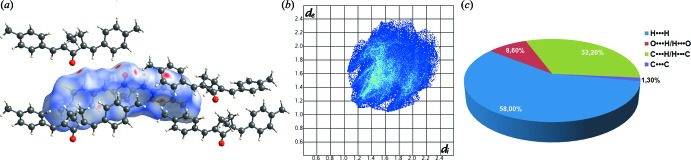
(*a*) View of the three-dimensional Hirshfeld surface mapped over *d*_norm_, (*b*) the two-dimensional fingerprint plot and (*c*) the relative contributions to the Hirshfeld surface area by the various close inter­molecular contacts in the structure of MBMCP.

The inter­molecular inter­actions were qu­anti­fied using Hirshfeld surface analysis (Fig. 4 ▸). The Hirshfeld surfaces of MBMCP, their associated two-dimensional fingerprint plots and relative contributions to the Hirshfeld surface area from the various close inter­molecular contacts were calculated using *CrystalExplorer* software (Wolff *et al.*, 2007 ▸). The analysis of inter­mol­ecular inter­actions through the mapping of *d*_norm_ compares the contact distances *d*_i_ and *d*_e_ from the Hirshfeld surface to the nearest atom inside and outside, respectively, with their respective van der Waals radii. The red regions represent contacts shorter than the sum of van der Waals radii, white regions represent inter­molecular distances equal to van der Waals contacts and blue regions represent contacts longer than van der Waals radii.

**Figure 5:**
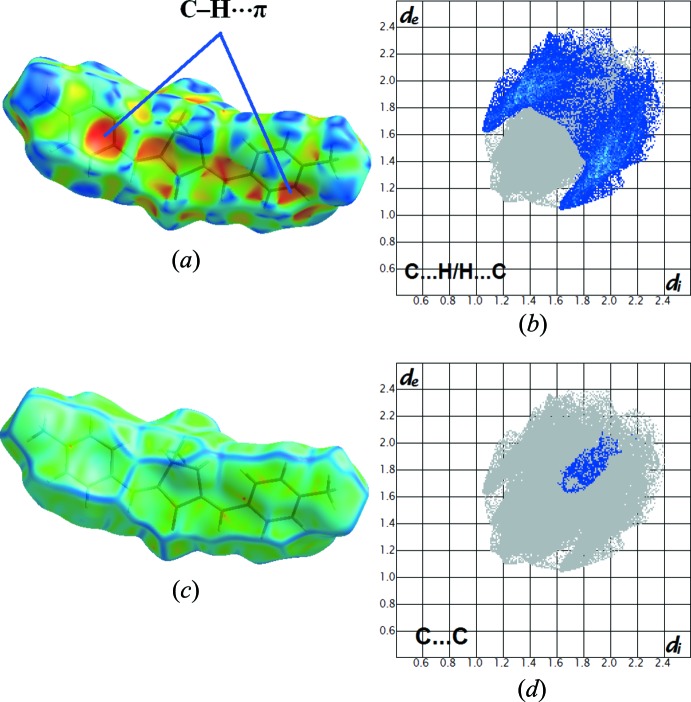
(*a*) The Hirshfeld surface mapped over shape-index, (*b*) the two-dimensional fingerprint plot for the H⋯C/C⋯H inter­actions, (*c*) the Hirshfeld surface mapped over curvedness and (*d*) the two-dimensional fingerprint plot for the C⋯C inter­actions in the title compound.

As expected in organic compounds, the shortest and most abundant contacts for MBMCP are the H⋯H inter­molecular inter­actions with a contribution to the Hirshfeld surface of 58%. The C⋯H contacts, which refer to the C—H⋯π inter­actions described previously, contribute 32.2% of the Hirshfeld surfaces. On the shape-index surface, the C—H⋯π inter­actions are clearly observed as red regions over the aromatic rings. The shape-index is in agreement with the 2D fingerprint plot, in which these inter­actions appear as two broad spikes both having *d*_e_ + *d*_i_ ∼2.7 Å (Fig. 5 ▸). The large flat region, delineated by a blue outline on the curvedness of MBMCP reveals that π–π stacking inter­actions are absent.

## Anti­bacterial activity   

The anti­bacterial activity of MBMCP was assayed *in vitro* against *Escherchia coli*, *Staphyococcus aureus, Salmonella typhi* and *Bacillus subtilis via* an agar cup-plate diffusion method (Barry, 1976 ▸; Ponce *et al.*, 2003 ▸). The bacteria tests were sub-cultured in Mueller–Hinton broth, from which 1 mL of cell suspension was taken and the optical density was adjusted to 0.5. The suspension was then spread as a thin film over the Mueller–Hinton agar plates. The synthetic compound was loaded onto discs with concentrations of 0.2, 0.3, 0.4 and 0.5 µg mL^−1^ and air-dried. The dry discs were placed on the inoculated Mueller–Hinton agar plates and incubated at 310 K for 48 h. A penicillin disc (10 µg per disc) was used as the standard. A disc of 150 µl of DMSO served as the control. After incubation, the zone of inhibition (in mm) was measured and compared with that of penicillin for each concentration.

The anti­bacterial screening results are collected in Table 2 ▸. They reveal that the produced compound shows an anti­bacterial activity at MIC = 0.5 µg mL^−1^ towards all the bacterial strains, but differs from one strain to another when comparing the zones of inhibition (mm). It exhibits moderate and promising activities against *Staphylococcus aureus* (27 mm) and *Bacillus subtilis* (14 mm). However, it shows low activities against *Escherchia coli* (7 mm) and *Salmonella typhi* (12 mm), which probably demonstrate that the produced compound exhibits a specific effect on those microorganisms.

## Database survey   

A search of the Cambridge Structural Database, CSD (Version 5.38; *ConQuest* 1.19; Groom *et al.*, 2016 ▸) revealed 20 derivatives of bis­(benzyl­idene)cyclo­penta­none. The variety of compounds reported in the literature (Kawamata *et al.*, 1998 ▸; Nakhaei *et al.*, 2017 ▸) is due to substitution on the phenyl rings and/or on the cyclo­penta­none by different functional groups, such as hy­droxy, meth­oxy, chlorine, fluorine *etc*., and also by radicals. Cyclic conjugated *bis*(benzyl­idene)ketones have been reported to exhibit potent anti-inflammatory, anti­bacterial and anti­oxidant activity (Shetty *et al.*, 2015 ▸). *E*,*E*-2,5-di­benzyl­idene-3-methyl­cyclo­penta­none **(**DBMCP**)** is the nearest analogue to MBMCP. This mol­ecule, like that of the title compound, exhibits a twisted five-membered ring, conveying modest non-planarity to the overall mol­ecular shape with a maximum deviation from the mean plane of 0.44 Å (Theocharis *et al.*, 1984 ▸). The basic skeleton of this compound family, *E*,*E*-2,5-di­benzyl­idene­cyclo­penta­none (DBCP), has been isolated in two polymorphic forms, exhibiting two different but nearly superimposable conformations (Arshad *et al.*, 2014 ▸). The previously reported polymorph I crystallizes in the ortho­rhom­bic *C*222_1_ space group (Theocharis *et al.*, 1984 ▸), while the second form crystallizes in the monoclinic *P*2_1_ space group. Both forms pack as supra­molecular chains mainly stabilized by C—H⋯O, π–π and C—H⋯π inter­actions and forming sheet-like multilayered structures.

## Synthesis and crystallization   

A mixture of 4-methyl­benzaldehyde (20 mmol, 2 eq.) and 3-methyl­cyclo­penta­none (10 mmol, 1 eq.) were dissolved in ethanol (15 mL) into a flask (simple necked, round bottomed), and the solution was stirred for a few minutes at 273 K (ice bath). A solution of NaOH (10 mL, 40%) was added dropwise over several minutes into this mixture. The resulting mixture was stirred for 4 h approximately at room temperature. The obtained yellow precipitate was then filtered, washed with HCl (0.1 *N*) and cold water and then dried. The pure product was crystallized from ethanol solution at room temperature in 75% yield. Single crystals for X-ray diffraction were grown by slow solvent evaporation from a solution in ethanol.

The FT–IR spectrum of the compound was measured by the KBr pellet technique in the range of 4000–400 cm^−1^, with a Nexus Nicolet FT–IR spectrometer at a resolution of 2 cm^−1^. A Bruker Optik GmbH system was utilized to measure the Raman spectrum of the powder compound. A class 4 laser Raman spectrometer of 532 nm excitation from a diode laser (3B) was used with 2 cm^−1^ resolution within the spectroscopic range 3500–0 cm^−1. 1^H and ^13^C NMR spectra were qu­anti­fied with CDCl_3_ using a (400.13 MHz in 1H) Avance 400 Bruker spectrometer with TMS as inter­nal standard.

^1^H NMR (400 MHz, CDCl_3_) δ (ppm): 1.23 (*d*, 1H, CH_3_), 1.54 (*s*, 2H, H_2_O), 2.39 (*s*, 6H, 2CH_3_), 2.76 (*dd*, 1H_cycle_), 3.18 (*ddd*, 1H_cycle_), 3.66 (*m*, 1H_cycle_), 7.22 (*d*, 2H_ar­yl_), 7.25 (*d*, 2H_ar­yl_), 7.47 (*d*, 3H_ar­yl_), 7.51 (*s*, 1H_ethyl­enic_), 7.53 (*s*, 1H_ethyl­enic_), and 7.66 (t, 1H_ar­yl_).

^13^C NMR (75.46 MHz, CDCl_3_) δ (ppm): 21.03 (CH_3_), 23.47 (CH_3_), 23.49 (CH_3_), 32.01 (CH_cycle_), 35.98 (CH_2cycle_), 129.51 (2C—Ar), 129.62 (2C—Ar), 130.71 (2C—Ar), 130.79 (2C—Ar), 133.37 (2C—Ar), 134.38 (2C—Ar), 135.57 (CH=C_cycle_), 139.72 (C_cycle_=CH), 139.76 (C_cycle_=CH), 142.51 (CH=C_cycle_), and 196.46 (C=O).

Fourier–transform infrared (FT–IR) spectroscopy (KBr, cm^−1^): 3056–3022 (CH _aromatic_), 2863 (CH _alyphatic_), 1670 (C=O), 1616 and 1596 (C=C), and 1590.90, 1545.45 (C=C of aromatic rings).

Raman spectroscopy with 1000–1670 Hz frequency range for the C—C, C=C and C=O bonds. In addition, 2800–3156 Hz frequency domain for C—H bonds. Ultraviolet (UV) spectroscopy [EtOH, λ (nm)]: 208, 237 assignable to π–π, and 364, 376 assignable to π–π* transitions.

## Refinement   

Crystal data, data collection and structure refinement details are summarized in Table 3 ▸. H atoms were included in their calculated positions and refined using the riding-atom approximation: C—H = 0.96 Å (methyl CH_3_) and 0.93 Å (ar­yl), with *U*_iso_(H) = 1.5*U*_eq_(C) for methyl H atoms and 1.2*U*_eq_(C) for all other H atoms.

## Supplementary Material

Crystal structure: contains datablock(s) I. DOI: 10.1107/S2056989019003827/fy2134sup1.cif

Structure factors: contains datablock(s) I. DOI: 10.1107/S2056989019003827/fy2134Isup2.hkl

Supporting information file. DOI: 10.1107/S2056989019003827/fy2134Isup3.cml

CCDC reference: 1515960

Additional supporting information:  crystallographic information; 3D view; checkCIF report

## Figures and Tables

**Table 1:** Hydrogen-bond geometry (Å, °) *Cg*1 and *Cg*2 are the centroids of the C2–C7 and C17–C22 rings, respectively.

*D*—H⋯*A*	*D*—H	H⋯*A*	*D*⋯*A*	*D*—H⋯*A*
C1—H1*C*⋯*Cg*1^i^	0.98	2.84	3.728 (3)	152
C4—H4⋯*Cg*2^ii^	0.95	2.75	3.574 (2)	146

Symmetry codes: (i) 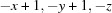; (ii) 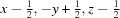.

**Table 2:** Anti­bacterial screening results (zone of inibition, mm)

Compound	*Escherchia coli*	*Salmonella typhi*	*Staphylococcus aureus*	*Bacillus subtilis*
MBMCP	07	12	27	14
Penecillin	18	25	40	17
DMSO	–	–	–	–

(-) No anti­bacterial activity

**Table 3:** Experimental details

Crystal data	
Chemical formula	C_22_H_22_O
*M*_r_	302.39
Crystal system, space group	Monoclinic, *P*2_1_/*n*
Temperature (K)	150
*a*, *b*, *c* (Å)	9.8037 (9), 8.9815 (9), 18.8946 (17)
β (°)	90.985 (4)
*V* (Å^3^)	1663.5 (3)
*Z*	4
Radiation type	Mo *K*α
μ (mm^−1^)	0.07
Crystal size (mm)	0.52 × 0.28 × 0.06

Data collection	
Diffractometer	Bruker D8 VENTURE
Absorption correction	Multi-scan (*SADABS*; Bruker, 2014 ▸)
*T*_min_, *T*_max_	0.735, 0.996
No. of measured, independent and observed [*I* > 2σ(*I*)] reflections	15325, 3808, 2471
*R*_int_	0.115

Refinement	
*R*[*F* ^2^ > 2σ(*F* ^2^)], *wR*(*F* ^2^), *S*	0.070, 0.171, 1.12
No. of reflections	3808
No. of parameters	211
H-atom treatment	H-atom parameters constrained
Δρ_max_, Δρ_min_ (e Å^−3^)	0.30, −0.26

Computer programs: *APEX3* (Bruker, 2015 ▸), *SAINT* (Bruker, 2014 ▸), *SHELXT* (Sheldrick, 2015*a* ▸), *SHELXL2014* (Sheldrick, 2015*b* ▸), *ORTEP-3 for Windows* (Farrugia, 2012 ▸), *DIAMOND* (Brandenburg & Berndt, 2001 ▸) and *CRYSCALc* (T. Roisnel, local program, version of 2015).

## supplementary crystallographic information

### Crystal data

**Table:**

C_22_H_22_O	*F*(000) = 648
*M**_r_* = 302.39	*D*_x_ = 1.207 Mg m^−^^3^
Monoclinic, *P*2_1_/*n*	Mo *K*α radiation, λ = 0.71073 Å
*a* = 9.8037 (9) Å	Cell parameters from 2848 reflections
*b* = 8.9815 (9) Å	θ = 2.4–27.4°
*c* = 18.8946 (17) Å	µ = 0.07 mm^−^^1^
β = 90.985 (4)°	*T* = 150 K
*V* = 1663.5 (3) Å^3^	Thick plate, colourless
*Z* = 4	0.52 × 0.28 × 0.06 mm

### Data collection

**Table:**

Bruker D8 VENTURE diffractometer	3808 independent reflections
Radiation source: Incoatec microfocus sealed tube	2471 reflections with *I* > 2σ(*I*)
Multilayer monochromator	*R*_int_ = 0.115
rotation images scans	θ_max_ = 27.5°, θ_min_ = 3.1°
Absorption correction: multi-scan (SADABS; Bruker, 2014)	*h* = −12→12
*T*_min_ = 0.735, *T*_max_ = 0.996	*k* = −11→10
15325 measured reflections	*l* = −24→24

### Refinement

**Table:**

Refinement on *F*^2^	Primary atom site location: structure-invariant direct methods
Least-squares matrix: full	Secondary atom site location: difference Fourier map
*R*[*F*^2^ > 2σ(*F*^2^)] = 0.070	Hydrogen site location: inferred from neighbouring sites
*wR*(*F*^2^) = 0.171	H-atom parameters constrained
*S* = 1.12	*w* = 1/[σ^2^(*F*_o_^2^) + (0.0652*P*)^2^ + 0.431*P*] where *P* = (*F*_o_^2^ + 2*F*_c_^2^)/3
3808 reflections	(Δ/σ)_max_ = 0.005
211 parameters	Δρ_max_ = 0.30 e Å^−^^3^
0 restraints	Δρ_min_ = −0.26 e Å^−^^3^

### Special details

**Table:**

Geometry. All esds (except the esd in the dihedral angle between two l.s. planes) are estimated using the full covariance matrix. The cell esds are taken into account individually in the estimation of esds in distances, angles and torsion angles; correlations between esds in cell parameters are only used when they are defined by crystal symmetry. An approximate (isotropic) treatment of cell esds is used for estimating esds involving l.s. planes.
Refinement. Refinement of F^2^ against ALL reflections. The weighted R-factor wR and goodness of fit S are based on F^2^, conventional R-factors R are based on F, with F set to zero for negative F^2^. The threshold expression of F^2^ > 2sigma(F^2^) is used only for calculating R-factors(gt) etc. and is not relevant to the choice of reflections for refinement. R-factors based on F^2^ are statistically about twice as large as those based on F, and R- factors based on ALL data will be even larger.

### Fractional atomic coordinates and isotropic or equivalent isotropic displacement parameters (Å^2^)

**Table:**

	*x*	*y*	*z*	*U*_iso_*/*U*_eq_	
C1	0.6385 (3)	0.4045 (3)	−0.09361 (12)	0.0385 (6)	
H1A	0.6054	0.3291	−0.1273	0.058*	
H1B	0.7347	0.4259	−0.1025	0.058*	
H1C	0.5848	0.4958	−0.0994	0.058*	
C2	0.6242 (2)	0.3472 (3)	−0.01957 (12)	0.0274 (5)	
C3	0.5158 (2)	0.2568 (3)	−0.00074 (13)	0.0357 (6)	
H3	0.4503	0.2287	−0.0359	0.043*	
C4	0.5009 (2)	0.2070 (3)	0.06766 (12)	0.0316 (6)	
H4	0.4260	0.1443	0.0785	0.038*	
C5	0.5940 (2)	0.2468 (2)	0.12164 (11)	0.0227 (5)	
C6	0.7056 (2)	0.3345 (3)	0.10266 (12)	0.0286 (5)	
H6	0.7722	0.3611	0.1375	0.034*	
C7	0.7195 (2)	0.3824 (3)	0.03386 (12)	0.0308 (6)	
H7	0.7964	0.4414	0.0223	0.037*	
C8	0.5659 (2)	0.1961 (3)	0.19360 (12)	0.0242 (5)	
H8	0.4994	0.1195	0.1962	0.029*	
C9	0.6180 (2)	0.2391 (2)	0.25660 (12)	0.0226 (5)	
C10	0.5636 (2)	0.1772 (2)	0.32316 (12)	0.0232 (5)	
O11	0.49025 (16)	0.06702 (19)	0.32852 (8)	0.0328 (4)	
C12	0.6133 (2)	0.2716 (2)	0.38293 (12)	0.0228 (5)	
C13	0.6898 (2)	0.4010 (2)	0.35203 (11)	0.0244 (5)	
H13A	0.7731	0.4227	0.3805	0.029*	
H13B	0.6318	0.4913	0.3506	0.029*	
C14	0.7269 (2)	0.3520 (2)	0.27653 (12)	0.0235 (5)	
H14	0.7247	0.4386	0.2433	0.028*	
C15	0.8679 (2)	0.2773 (3)	0.27741 (13)	0.0315 (6)	
H15A	0.8700	0.1980	0.3130	0.047*	
H15B	0.9381	0.3515	0.2891	0.047*	
H15C	0.8856	0.2349	0.2307	0.047*	
C16	0.5869 (2)	0.2357 (2)	0.44987 (11)	0.0236 (5)	
H16	0.5378	0.1453	0.4553	0.028*	
C17	0.6207 (2)	0.3119 (2)	0.51624 (11)	0.0230 (5)	
C18	0.6069 (2)	0.2345 (3)	0.57982 (12)	0.0262 (5)	
H18	0.5783	0.1335	0.5787	0.031*	
C19	0.6338 (2)	0.3014 (3)	0.64411 (12)	0.0288 (6)	
H19	0.6255	0.2451	0.6864	0.035*	
C20	0.6730 (2)	0.4508 (3)	0.64823 (12)	0.0269 (5)	
C21	0.6866 (2)	0.5284 (3)	0.58517 (12)	0.0274 (5)	
H21	0.7138	0.6299	0.5866	0.033*	
C22	0.6614 (2)	0.4612 (3)	0.52014 (12)	0.0247 (5)	
H22	0.6718	0.5170	0.4779	0.030*	
C23	0.6981 (3)	0.5255 (3)	0.71853 (13)	0.0394 (7)	
H23A	0.7168	0.6314	0.7110	0.059*	
H23B	0.7766	0.4790	0.7425	0.059*	
H23C	0.6172	0.5147	0.7478	0.059*	

### Atomic displacement parameters (Å^2^)

**Table:**

	*U*^11^	*U*^22^	*U*^33^	*U*^12^	*U*^13^	*U*^23^
C1	0.0416 (15)	0.0411 (16)	0.0330 (14)	−0.0006 (12)	0.0010 (11)	0.0032 (12)
C2	0.0267 (12)	0.0281 (13)	0.0273 (12)	0.0035 (10)	0.0014 (10)	−0.0004 (10)
C3	0.0260 (12)	0.0494 (17)	0.0314 (14)	−0.0082 (11)	−0.0056 (10)	−0.0007 (12)
C4	0.0218 (12)	0.0392 (15)	0.0338 (14)	−0.0112 (10)	0.0002 (10)	−0.0009 (11)
C5	0.0189 (10)	0.0199 (11)	0.0293 (12)	0.0013 (9)	0.0013 (9)	0.0003 (9)
C6	0.0218 (11)	0.0329 (14)	0.0309 (13)	−0.0075 (10)	−0.0032 (9)	−0.0017 (11)
C7	0.0279 (12)	0.0310 (14)	0.0337 (13)	−0.0112 (10)	0.0049 (10)	0.0000 (11)
C8	0.0168 (10)	0.0214 (12)	0.0346 (13)	0.0001 (9)	0.0000 (9)	0.0012 (10)
C9	0.0180 (10)	0.0180 (11)	0.0318 (12)	0.0041 (8)	0.0001 (9)	0.0022 (10)
C10	0.0198 (11)	0.0198 (11)	0.0299 (12)	0.0018 (9)	−0.0011 (9)	0.0049 (10)
O11	0.0343 (9)	0.0284 (9)	0.0357 (10)	−0.0127 (8)	−0.0001 (7)	0.0056 (8)
C12	0.0191 (10)	0.0178 (11)	0.0314 (12)	0.0040 (8)	−0.0008 (9)	0.0037 (10)
C13	0.0240 (11)	0.0192 (12)	0.0298 (12)	−0.0016 (9)	−0.0013 (9)	0.0019 (10)
C14	0.0202 (11)	0.0203 (12)	0.0299 (12)	−0.0027 (9)	0.0016 (9)	0.0031 (10)
C15	0.0212 (11)	0.0354 (15)	0.0380 (14)	−0.0036 (10)	−0.0009 (10)	−0.0031 (11)
C16	0.0205 (11)	0.0180 (11)	0.0323 (13)	0.0033 (9)	0.0017 (9)	0.0041 (10)
C17	0.0168 (10)	0.0234 (12)	0.0289 (12)	0.0040 (9)	0.0029 (9)	0.0053 (10)
C18	0.0208 (11)	0.0248 (13)	0.0331 (13)	0.0035 (9)	0.0032 (9)	0.0065 (10)
C19	0.0222 (11)	0.0374 (14)	0.0271 (13)	0.0108 (10)	0.0050 (9)	0.0104 (11)
C20	0.0164 (10)	0.0387 (14)	0.0258 (12)	0.0111 (10)	0.0011 (9)	−0.0002 (11)
C21	0.0223 (11)	0.0247 (13)	0.0352 (14)	0.0049 (9)	0.0017 (10)	0.0018 (10)
C22	0.0227 (11)	0.0263 (13)	0.0252 (12)	0.0043 (9)	0.0005 (9)	0.0061 (10)
C23	0.0380 (14)	0.0501 (17)	0.0302 (14)	0.0100 (12)	0.0005 (11)	−0.0021 (12)

### Geometric parameters (Å, º)

**Table:**

C1—C2	1.499 (3)	C13—H13A	0.9900
C1—H1A	0.9800	C13—H13B	0.9900
C1—H1B	0.9800	C14—C15	1.536 (3)
C1—H1C	0.9800	C14—H14	1.0000
C2—C3	1.388 (3)	C15—H15A	0.9800
C2—C7	1.400 (3)	C15—H15B	0.9800
C3—C4	1.378 (3)	C15—H15C	0.9800
C3—H3	0.9500	C16—C17	1.462 (3)
C4—C5	1.404 (3)	C16—H16	0.9500
C4—H4	0.9500	C17—C18	1.396 (3)
C5—C6	1.399 (3)	C17—C22	1.401 (3)
C5—C8	1.464 (3)	C18—C19	1.377 (3)
C6—C7	1.379 (3)	C18—H18	0.9500
C6—H6	0.9500	C19—C20	1.398 (4)
C7—H7	0.9500	C19—H19	0.9500
C8—C9	1.344 (3)	C20—C21	1.389 (3)
C8—H8	0.9500	C20—C23	1.504 (3)
C9—C10	1.483 (3)	C21—C22	1.387 (3)
C9—C14	1.515 (3)	C21—H21	0.9500
C10—O11	1.228 (3)	C22—H22	0.9500
C10—C12	1.488 (3)	C23—H23A	0.9800
C12—C16	1.335 (3)	C23—H23B	0.9800
C12—C13	1.506 (3)	C23—H23C	0.9800
C13—C14	1.543 (3)		
			
C2—C1—H1A	109.5	H13A—C13—H13B	108.8
C2—C1—H1B	109.5	C9—C14—C15	109.94 (18)
H1A—C1—H1B	109.5	C9—C14—C13	104.13 (17)
C2—C1—H1C	109.5	C15—C14—C13	109.97 (18)
H1A—C1—H1C	109.5	C9—C14—H14	110.9
H1B—C1—H1C	109.5	C15—C14—H14	110.9
C3—C2—C7	116.8 (2)	C13—C14—H14	110.9
C3—C2—C1	121.6 (2)	C14—C15—H15A	109.5
C7—C2—C1	121.6 (2)	C14—C15—H15B	109.5
C4—C3—C2	121.6 (2)	H15A—C15—H15B	109.5
C4—C3—H3	119.2	C14—C15—H15C	109.5
C2—C3—H3	119.2	H15A—C15—H15C	109.5
C3—C4—C5	121.4 (2)	H15B—C15—H15C	109.5
C3—C4—H4	119.3	C12—C16—C17	130.9 (2)
C5—C4—H4	119.3	C12—C16—H16	114.6
C6—C5—C4	117.3 (2)	C17—C16—H16	114.6
C6—C5—C8	125.0 (2)	C18—C17—C22	117.6 (2)
C4—C5—C8	117.7 (2)	C18—C17—C16	118.7 (2)
C7—C6—C5	120.5 (2)	C22—C17—C16	123.6 (2)
C7—C6—H6	119.7	C19—C18—C17	121.5 (2)
C5—C6—H6	119.7	C19—C18—H18	119.3
C6—C7—C2	122.3 (2)	C17—C18—H18	119.3
C6—C7—H7	118.9	C18—C19—C20	121.1 (2)
C2—C7—H7	118.9	C18—C19—H19	119.5
C9—C8—C5	131.2 (2)	C20—C19—H19	119.5
C9—C8—H8	114.4	C21—C20—C19	117.7 (2)
C5—C8—H8	114.4	C21—C20—C23	121.1 (2)
C8—C9—C10	120.4 (2)	C19—C20—C23	121.2 (2)
C8—C9—C14	131.9 (2)	C22—C21—C20	121.6 (2)
C10—C9—C14	107.62 (18)	C22—C21—H21	119.2
O11—C10—C9	126.3 (2)	C20—C21—H21	119.2
O11—C10—C12	125.5 (2)	C21—C22—C17	120.6 (2)
C9—C10—C12	108.17 (19)	C21—C22—H22	119.7
C16—C12—C10	121.0 (2)	C17—C22—H22	119.7
C16—C12—C13	131.3 (2)	C20—C23—H23A	109.5
C10—C12—C13	107.73 (18)	C20—C23—H23B	109.5
C12—C13—C14	105.37 (18)	H23A—C23—H23B	109.5
C12—C13—H13A	110.7	C20—C23—H23C	109.5
C14—C13—H13A	110.7	H23A—C23—H23C	109.5
C12—C13—H13B	110.7	H23B—C23—H23C	109.5
C14—C13—H13B	110.7		
			
C7—C2—C3—C4	1.4 (4)	C16—C12—C13—C14	160.9 (2)
C1—C2—C3—C4	−178.5 (2)	C10—C12—C13—C14	−19.5 (2)
C2—C3—C4—C5	0.8 (4)	C8—C9—C14—C15	−88.7 (3)
C3—C4—C5—C6	−2.4 (4)	C10—C9—C14—C15	93.9 (2)
C3—C4—C5—C8	176.5 (2)	C8—C9—C14—C13	153.5 (2)
C4—C5—C6—C7	1.9 (3)	C10—C9—C14—C13	−23.9 (2)
C8—C5—C6—C7	−176.9 (2)	C12—C13—C14—C9	26.3 (2)
C5—C6—C7—C2	0.3 (4)	C12—C13—C14—C15	−91.4 (2)
C3—C2—C7—C6	−2.0 (4)	C10—C12—C16—C17	−178.0 (2)
C1—C2—C7—C6	178.0 (2)	C13—C12—C16—C17	1.6 (4)
C6—C5—C8—C9	13.9 (4)	C12—C16—C17—C18	−166.3 (2)
C4—C5—C8—C9	−164.9 (2)	C12—C16—C17—C22	16.4 (4)
C5—C8—C9—C10	175.1 (2)	C22—C17—C18—C19	−0.8 (3)
C5—C8—C9—C14	−2.0 (4)	C16—C17—C18—C19	−178.30 (19)
C8—C9—C10—O11	14.6 (3)	C17—C18—C19—C20	1.5 (3)
C14—C9—C10—O11	−167.6 (2)	C18—C19—C20—C21	−1.3 (3)
C8—C9—C10—C12	−165.43 (19)	C18—C19—C20—C23	178.0 (2)
C14—C9—C10—C12	12.3 (2)	C19—C20—C21—C22	0.4 (3)
O11—C10—C12—C16	4.3 (3)	C23—C20—C21—C22	−178.8 (2)
C9—C10—C12—C16	−175.62 (19)	C20—C21—C22—C17	0.2 (3)
O11—C10—C12—C13	−175.4 (2)	C18—C17—C22—C21	0.0 (3)
C9—C10—C12—C13	4.7 (2)	C16—C17—C22—C21	177.3 (2)

### Hydrogen-bond geometry (Å, º)

*Cg*1 and *Cg*2 are the centroids of the C2–C7 and C17–C22 rings, respectively.

**Table:**

*D*—H···*A*	*D*—H	H···*A*	*D*···*A*	*D*—H···*A*
C1—H1*C*···*Cg*1^i^	0.98	2.84	3.728 (3)	152
C4—H4···*Cg*2^ii^	0.95	2.75	3.574 (2)	146

Symmetry codes: (i) −*x*+1, −*y*+1, −*z*; (ii) *x*−1/2, −*y*+1/2, *z*−1/2.
